# Detecting GPC3-Expressing Hepatocellular Carcinoma with L5 Peptide-Guided Pretargeting Approach: In Vitro and In Vivo MR Imaging Experiments

**DOI:** 10.1155/2018/9169072

**Published:** 2018-09-10

**Authors:** Weiyue Li, Xiang Xiao, Xiaodan Li, Yikai Xu, Lichao Ma, Liuji Guo, Chenggong Yan, Yuankui Wu

**Affiliations:** ^1^Department of Medical Imaging Centre, Nanfang Hospital, Southern Medical University, Guangzhou, China; ^2^Department of Radiology, Shenzhen People's Hospital, Shenzhen, China

## Abstract

**Objective:**

To investigate the potential of L5 peptide-guided pretargeting approach to identify GPC3-expressing hepatocellular carcinoma (HCC) using ultrasmall superparamagnetic iron oxide (USPIO) as the MR probe.

**Methods:**

Immunofluorescence with carboxyfluorescein- (FAM-) labeled L5 peptide was performed in HepG2 cells. Polyethylene glycol-modified USPIO (PEG-USPIO) and its conjugation with streptavidin (SA-PEG-USPIO) were synthesized, and their hydrodynamic diameters, zeta potential, T_2_ relaxivity, and cytotoxicity were measured. In vitro and in vivo two-step pretargeting MR imaging was performed on HepG2 cells and tumor-bearing mice after the administration of biotinylated L5 peptide (first step), followed by SA-PEG-USPIO (second step). Prussian blue staining was performed to assess iron deposition in tumors.

**Results:**

The high specificity of L5 peptide for GPC3 was demonstrated. Generation of SA-PEG-USPIO nanoparticles with good biocompatibility (an average hydrodynamic diameter of 35.97 nm and a zeta potential of −7.91 mV), superparamagnetism (*R*_2_ = 0.1039 × 10^3^ mM^−1^s^−1^), and low toxicity was achieved. The pretargeting group showed more enhancement than the nonpretargeting group both in vitro (60% vs 20%, *P* < 0.05) and in vivo (32% vs 6%, *P* < 0.001). Substantial iron deposition was only observed in HepG2 cells and tumors in the pretargeting group.

**Conclusion:**

L5 peptide-guided, two-step pretargeting approach with USPIO as the MR imaging probe is a lucrative strategy to specifically identify GPC3-expressing HCC.

## 1. Introduction

Hepatocellular carcinoma (HCC) is a common human malignancy that affects diverse populations worldwide [1]. Accurate diagnosis plays a vital role in the management of patients with HCC. As the mainstay imaging modality, conventional contrast-enhanced magnetic resonance (MR) imaging suffers from low specificity for HCC [2]. A variety of MR imaging technologies have been explored, such as perfusion MR imaging, diffusion-weighted MR imaging, and MR imaging with superparamagnetic iron oxide (SPIO), ultrasmall superparamagnetic iron oxide (USPIO), or hepatobiliary agents. While these methods offer an increase in the ability to identify HCC, there is still much room for improvement [3–6].

Molecular MR imaging using magnetic nanoparticles (NPs) to specifically target tumor cells has been well documented as a good method to address issues with HCC diagnosis [7–9]. Glypican-3 (GPC3) may be the most promising among the specific molecular targets for HCC. It is highly expressed in most HCC cells while absent in normal liver parenchyma or benign liver lesions [10, 11], and it is more specific and sensitive than current biomarkers for small HCC, such as alpha-fetoprotein [11–14]. HCC foci were successfully identified using ^89^Zr coupled with an anti-GPC3 monoclonal antibody (aGPC3) as a PET probe [15]. MR imaging also can be used to detect HCC of very small size with USPIO-aGPC3 as a molecular probe [11]. Monoclonal antibodies (moAb) are widely used as ligands in molecular imaging for their targeting specificity and affinity for tumor biomarkers [16]. However, several inherent limitations of moAb, such as immunogenicity and high cost, severely hinder clinical translation of moAb-based approaches [17]. As an alternative to moAb, tumor homing peptides can be chosen as effective vectors to guide imaging probes to tumor cells [18–22]. Moreover, peptide ligands offer several advantages over moAb, such as fast blood clearance and excellent tissue penetration, which may produce a higher tumor-to-background ratio [23]. The L5 peptide consists of 14 amino acids (Arg-Leu-Asn-Val-Gly-Gly-Thr-Tyr-Phe-Leu-Thr-Thr-Arg-Gln) and has been proven able to specifically target GPC3-expressing HCC [22]. To the best of our knowledge, there is no published report on molecular MR imaging of HCC using L5 peptide targeting.

In this study, L5 peptide was utilized to bind GPC3 in HCC cells, and then it was connected to superparamagnetic NPs employing a two-step pretargeting protocol through biotin-avidin system. In vitro and in vivo MR imaging and histologic examination were performed to evaluate the specificity and feasibility of L5 peptide-based approach to identify GPC3-expressing HCC cells.

## 2. Materials and Methods

### 2.1. Materials

L5 peptide (Arg-Leu-Asn-Val-Gly-Gly-Thr-Tyr-Phe-Leu-Thr-Thr-Arg -Gln) was purchased from BambioCo, Ltd. (Xiamen, China). FAM, 4′,6-diamidino-2-phenylindole (DAPI), 0.1 M 2-morpholino-ethanesulfonic acid (MES) buffer solution, 1-ethyl-3-[3-dimethylaminopropyl] carbodiimide hydrochloride (EDC), sulfo-NHS, H_2_N-PEG-COOH, MTT, sulfo-NHS-LC-Biotin, and streptavidin (SA) were purchased from Sigma-Aldrich (St Louis, MO, USA). Dimethyl sulfoxide (DMSO), 4% paraformaldehyde, 2-mercaptoethanol, ethanolamine, and agarose were purchased from Aladdin-reagent (Shanghai, China). USPIO with carboxylate was purchased from Oneder Hightech Co. Ltd. (Beijing, China). All other chemicals were of analytical grade.

### 2.2. Cell Culture

HepG_2_ (human hepatocellular carcinoma cells expressing GPC3) and HL-7702 (human normal hepatocytes expressing little or no GPC3) cell lines were gifts from the Research Center of Clinical Medicine in Nanfang Hospital (Guangdong province, China). Both cell lines were routinely grown in Dulbecco's modified Eagle's medium supplemented with 10% (v/v) fetal bovine serum and 1% streptomycin-penicillin in a humidified incubator at 37°C with 5% CO_2_.

### 2.3. Synthesis of L5-FAM

L5 peptide (1.0 mg/ml, 1.0 ml) was combined with 15 *μ*g of EDC and 20 *μ*g of sulfo-NHS and stirred for 20 minutes to activate the carboxyl group of the peptide. A desalting column was used to remove excessive EDC and sulfo-NHS, followed by the addition of 0.2 mL of FAM (1.0 mg/ml). The mixture was stirred at 4°C in the dark for 12 hours. Excessive FAM was removed using a desalting column.

### 2.4. In Vitro Fluorescence Imaging

Fluorescence imaging was performed to verify the selective affinity of L5 peptide to GPC3-expressing HCC cells. HepG_2_ and HL-7702 cells were cultured on six-well chamber slides (5 × 10^5^ cells per slide) and grown for 24 hours at 37°C. Cells were washed with PBS three times and fixed in 4% paraformaldehyde/PBS solution for 30 minutes. The fixative was then removed, and cells were washed again with PBS three times. The slides were incubated with 0.1 mg/mL of L5-FAM or FAM in PBS/1% BSA, and then stained with DAPI for nuclear counterstaining. A blocking assay was conducted to evaluate L5 peptide specificity for GPC3, where the slides were incubated with 1 mg/mL of L5 peptide before adding 0.1 mg/mL of L5-FAM. Stained cells were observed with a fluorescence microscope (Eclipse TS100; Nikon, Tokyo, Japan).

### 2.5. Preparation of USPIO-PEG and SA-USPIO-PEG

A total of 10.0 mg of USPIO-COOH was dissolved in 10 mL of MES buffer (pH 5.5). EDC (0.6 mg) and sulfo-NHS (0.4 mg) were added to the mixture to activate the carboxyl. After 20 minutes, a desalting column was used to remove excessive EDC, and sulfo-NHS. H_2_N-PEG-COOH (0.6 g) was added to the solution while stirring, and excessive PEG was removed. Then, the PEG-USPIOs were concentrated by permanent magnet and dissolved in MES buffer.

EDC (2.0 mg) and sulfo-NHS (5.5 mg) were added to PEG-USPIO in 0.1 M MES buffer solution, and the reaction was maintained for 15 minutes at room temperature (RT) and then quenched with 2-mercaptoethanol. The solvent was removed by centrifugation at RT for 20 minutes at ×2500*g* (Millipore Amicon Ultra, Massachusetts, USA), and the resultant NPs were resuspended in PBS buffer solution and mixed with SA (3.0 mg SA) for 2 hours while stirring at RT before the reaction was stopped by adding ethanolamine. Finally, the solution was ultrafiltered by centrifugation, and the concentration was adjusted to 1.0 mg Fe/mL in PBS (pH 7.4).

### 2.6. NPs Characterization

The physical and morphological properties of PEG-USPIO and SA-USPIO-PEG in PBS were determined using a Zetasizer Nanoseries dynamic light scattering particle size analyzer (Malvern Zeta 3000HS, Worcestershire, UK) operating at 633.0 nm and 25.00 ± 0.05°C and transmission electron microscope (TEM; JEM-1230, JEOL, Tokyo, Japan), respectively.

### 2.7. Magnetic Property Measurements

The T_2_ relaxivity of PEG-USPIO and SA-USPIO-PEG was evaluated using a 3.0T MR system (Signa Excite; General Electric, Boston, USA) using T_2_ mapping sequence (TR = 2000 ms, TE = 20, 40, 60, 80 ms, FOV = 75 × 75 mm). Each was prepared in Fe concentrations of 0.04, 0.06, 0.08, 0.10, 0.12, 0.14, 0.16, 0.20, 0.40, and 0.60 mM. Images of the various solutions were analyzed by defining regions of interest (ROI) in each test tube. Relaxivity (*R*_2_) value was calculated through the curve fitting of T_2_ (per second) versus the Fe concentration (in micromoles).

### 2.8. Cytotoxicity Assay

In vitro cytotoxicity of the NPs (PEG-USPIO and SA-USPIO-PEG) was evaluated using the MTT assay in HL-7702 cells. In short, HL-7702 cells were seeded in 96-well plates at 6 × 10^3^ cells/well for 24 hours and then incubated with PEG-USPIO or SA-USPIO-PEG at different concentrations (0.4, 0.8, 1.2, 1.6, and 2.0 mM Fe) for 24 hours. Then, 20 *μ*L of MTT (5.0 mg/mL) was added to each well and incubated for 4 hours, followed by the addition of 150 *μ*L of DMSO. The OD_490_ value of each well was measured using a BIOTEK ELX800 microplate reader. The control group consisted only of cells and culture medium.

### 2.9. Biotinylation of L5

L5 peptides were biotinylated with Sulfo-NHS-LC-Biotin following the manufacturer's protocol. After purification with an Amicon Ultra-15 Centrifugal Filter Unit with a 1 kDa membrane from EMD Millipore (Billerica, MA, USA), the final biotin-peptide ratio was approximately 4, as determined by the HABA method.

### 2.10. In Vitro MR Imaging

**Figure 1:**
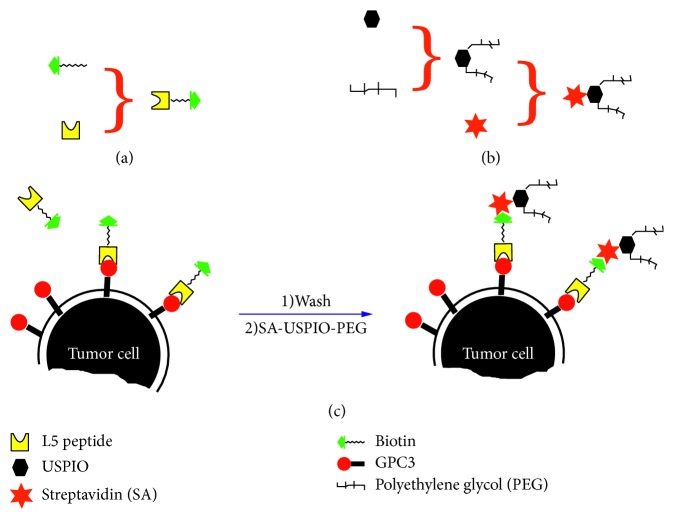
Schematic of NP synthesis and illustration of cell targeting. (a) Biotinylation of L5 peptide. (b) Synthesis of SA-USPIO-PEG. (c) Two-step pretargeting approach of cell labeling.

HepG2 and HL-7702 cells were seeded on 100 mm-diameter cell culture dishes and grown overnight. The pretargeting group (L5-BT-SA-USPIO-PEG), nonpretargeting group (SA-USPIO-PEG), and control group were set in the study. For the pretargeting group, 0.2 mg/mL of L5-BT was added to identify and bind to GPC3 molecules on tumor cells for 1 hour. Cells were then washed three times with PBS before incubation with SA-USPIO-PEG (Fe concentration of 1.8 mM) for 2 hours (Figure 1). For the nonpretargeting group, cells were incubated with SA-USPIO-PEG at the Fe concentration of 1.8 mM for 2 hours. Cells were left untreated in the control group. All groups were detached using ethylene diamine tetraacetic acid (EDTA; 1 : 5000; Invitrogen, Carlsbad, USA), centrifuged, resuspended in 1% agarose at a concentration of 0.5 × 107 cells per milliliter, and then transferred into 1.5 mL centrifuge tubes. Six replicates for each group were performed. T_2_-weighted images were performed with spin echo (SE) sequence (TR = 2500 ms, TE = 96 ms, NEX = 4, FOV = 75 × 75 mm, thickness = 2 mm, interval = 2 mm). The T2-weighted imaging (T2WI) signal intensity was normalized to that of 1% agarose. T_2_ color maps of HepG2 and HL-7702 cells in three different groups were obtained.

### 2.11. Prussian Blue Staining

According to standard clinical pathology protocols, both HepG2 and HL-7702 cells from the three groups (pretargeting, nonpretargeting, and control) were stained with Prussian blue after MR imaging [24].

### 2.12. In Vivo MR Imaging

All animal experiments were conducted in compliance with the regulations established by the Institutional Animal Care and Use Committee of Southern Medical University. Tumor xenografts with 1 × 10^7^ HepG2 cells were subcutaneously injected on the dorsum of 6- to 8-week-old Balb/c male mice (The Animal Center of Southern Medical University, Guangzhou, China). Three weeks after implantation (when the tumor reached ∼1.0 cm in diameter), animals were randomly separated into three groups: pretargeting group (L5-BT-SA-USPIO-PEG), nonpretargeting group (L5-SA-USPIO-PEG), and USPIO group for MR scan (*n*=8 per group).

Mice were subjected to tail vein injections with 50 *µ*g of biotinylated L5 peptide or L5 peptide over 2 minutes (first step). After 24 hours, SA-PEG-USPIO was injected via the tail vein at a dose of 80 *μ*mol Fe/kg (second step). For the USPIO group, only USPIO was administrated at the same dose as the pretargeting group. Mice were anesthetized with an intraperitoneal injection of pentobarbital sodium (50 mg/kg) 10 minutes before the MR studies. MR imaging was performed before and 1 hour after injection with a 3.0T MR system (Signa Excite) equipped with a mouse-imaging coil. Axial T_2_-weighted SE (TR/TE = 4000/85 ms, FOV = 12 cm, matrix = 320 × 224, NEX = 4, thickness/interval = 2.5/1.0 mm) was obtained.

In each T_2_-weighted image slice, a ROI was placed on the tumor and surrounding muscles in a blind manner by a radiologist (X. Li), and each average signal intensity (SI) was calculated. The enhanced ratio (ER) was calculated via the formula: ER = (SI_pre_−SI_post_)/SI_pre_.

### 2.13. Histologic Examination

Mice were sacrificed following MR imaging. Tumors were resected for histologic analysis, and Prussian blue staining was performed for the detection of iron in the tissue sections. Tumor morphology was verified by hematoxylin and eosin (HE) staining. Immunochemistry staining was performed to verify the expression of GPC3 protein in HepG2 xenografts. Sections were examined using a digital microscope (Olympus IX71; Tokyo, Japan). All pathological data were evaluated by a pathologist (Xiang Xiao) in a blind manner.

### 2.14. Statistical Analysis

All data were expressed as mean ± standard deviation. One-way analysis of variance and SNK were used to evaluate the differences of T2WI signal intensity among three groups for the in vitro MR imaging. Student's *t*-test or paired *t*-test was used for the comparison between groups of the in vivo MR imaging. All tests were performed using SPSS version 13.0 (IBM Corporation, Armonk, NY, USA). A statistically significant difference was defined when *P* < 0.05.

## 3. Results

### 3.1. In Vitro Fluorescence Imaging

**Figure 2:**
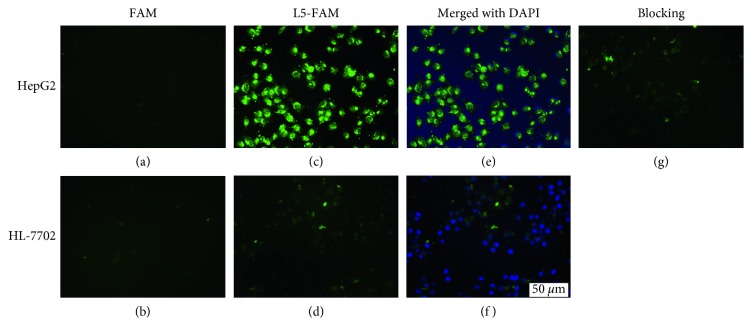
L5 peptide binding assays. HepG2 cells and HL-7702 cells were incubated with either FAM (a, b) or L5-FAM (c, d), respectively, which was mounted with DAPI-containing media (e, f). Excess L5 peptide blocked the binding of HepG2 cells with L5-FAM, exemplifying competitive binding and strong affinity of L5 peptide to GPC3 (g).

Cellular labeling with carboxyfluorescein (FAM) was visualized through fluorescence imaging. In the L5-FAM group, extensive cell membrane labeling occurred in HepG_2_ compared with HL-7702 cells. In the FAM group, neither HepG_2_ cells nor HL-7702 cells were labeled. In the blocking group, an excess of free L5 peptide precluded the binding of FAM-labeled L5 peptide to GPC3, resulting in a decreased fluorescent signal in HepG_2_ cells (Figure 2).

### 3.2. Characterization of PEG-USPIO and SA-USPIO-PEG

**Figure 3:**
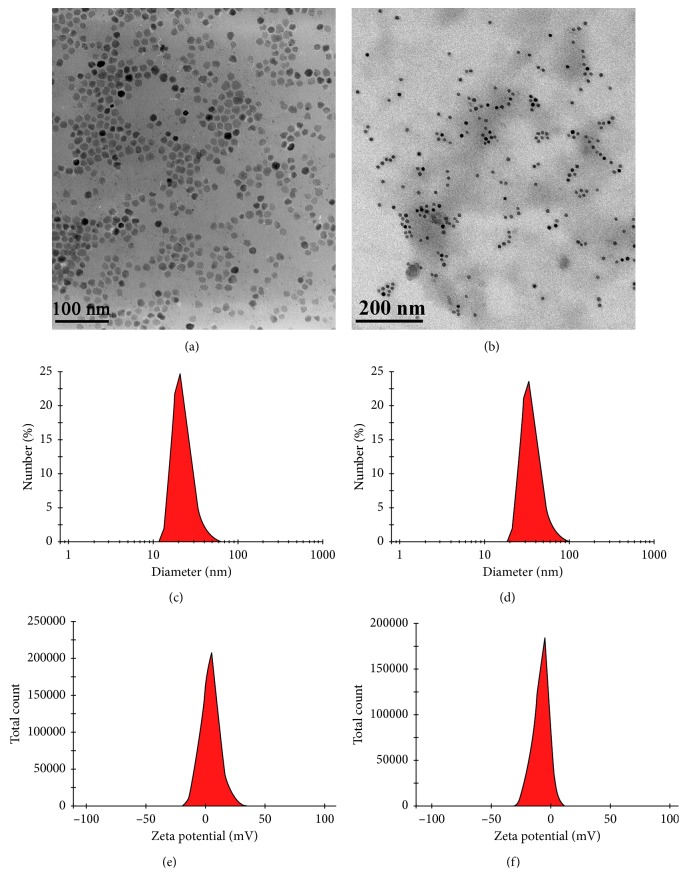
Physical and morphological properties of NPs. TEM picture of the USPIO (a) and SA-USPIO-PEG (b). Hydrodynamic size of PEG-USPIO (c) and SA-USPIO-PEG (d) in PBS, determined by dynamic light scattering. Zeta potential of PEG-USPIO (e) and SA-USPIO-PEG (f) at pH 7.0.

USPIO particles were uniformly distributed and had a core size of about 10–15 nm (Figure 3(a)). The morphology of SA-USPIO-PEG was shown in Figure 3(b). PEG-USPIO and SA-USPIO-PEG had an average hydrodynamic diameter of 22.73 nm and 35.97 nm, a polydispersity index of 0.207 and 0.169, and a zeta potential of 4.22 mV and −7.91 mV, respectively (Figures 3(c)–3(f)).

### 3.3. Magnetic Property Measurements

**Figure 4:**
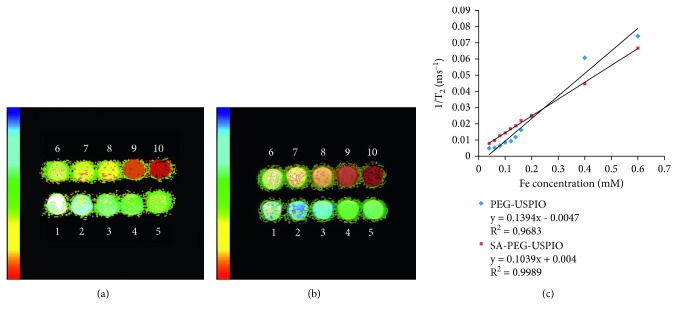
Magnetic property of NPs. The T_2_ color maps of PEG-USPIO (a) and SA-USPIO-PEG (b). Numbers 1–10 represent Fe concentrations ranging from 0.04 to 0.6 mM. *R*^2^ value curves of both NPs (c).

The pseudocolored images of T_2_ values illustrated that the color of the NPs deepened with an increase in Fe concentration (Figures 4(a) and 4(b)). *R*_2_ values were 0.1394 × 10^3^ mM^−1^s^−1^ and 0.1039 × 10^3^ mM^−1^s^−1^ for PEG-USPIO and SA-USPIO-PEG, respectively (Figure 4(c)).

### 3.4. Cytotoxicity Assay

**Figure 5:**
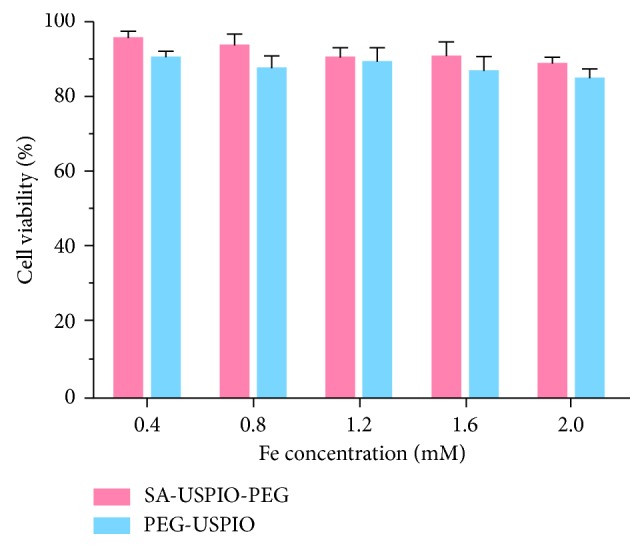
Cell viability of HL-7702 incubated with PEG-USPIO and SA-USPIO-PEG at various Fe concentrations.

To evaluate the cytotoxicity of nanoparticles, HL-7702 cells were incubated with PEG-USPIO and SA-USPIO-PEG for 24 hours, and then, cell viability was assessed via a methyl thiazoly tetrazolium (MTT) assay. As shown in Figure 5, cell viability did not significantly change with increasing Fe concentrations and still remained above 80% at the maximal Fe concentration. These results demonstrated that both NPs display low toxicity and may be biocompatible at the given Fe concentration range (0.4–2.0 mM).

### 3.5. In Vitro MR Imaging

**Figure 6:**
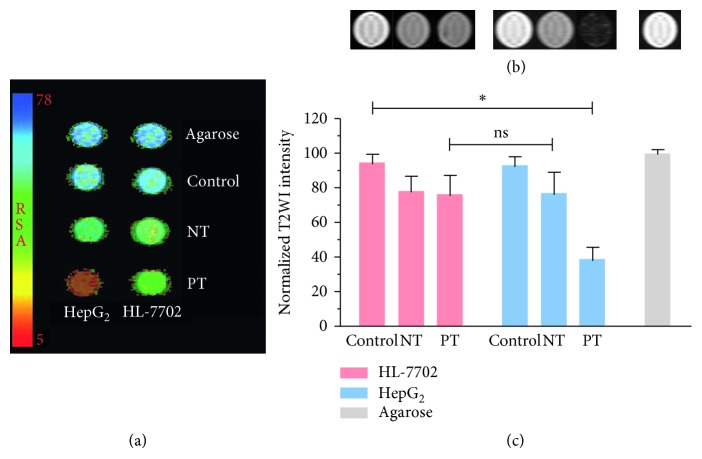
In vitro MR imaging of GPC3-targeted NPs uptake. (a) T_2_ color maps, (b) T2WI MR images, and (c) normalized T_2_ signal intensities. All demonstrated the most significant NPs uptake in HepG2 cells in the pretargeting group. PT, pretargeting group; NT, nonpretargeting group.

In vitro MR imaging was performed to test the feasibility of identifying GPC3-expressing HCC cells through L5 peptide-mediated pretargeting approach. Both the T_2_ color maps and T2WI images showed an approximate 60% decrease in signal intensity in HepG_2_ cells in the pretargeting group (Figures 6 and 6), compared to about a 20% decrease in the nonpretargeting group. A quantitative analysis showed that the normalized signal intensity of HepG_2_ cells in the pretargeting group was lower than that of any other group (*P* < 0.05), while the difference between the nonpretargeting and pretargeting groups in HL-7702 cells was not statistically significant (Figure 6).

### 3.6. In Vivo MR Imaging

**Figure 7:**
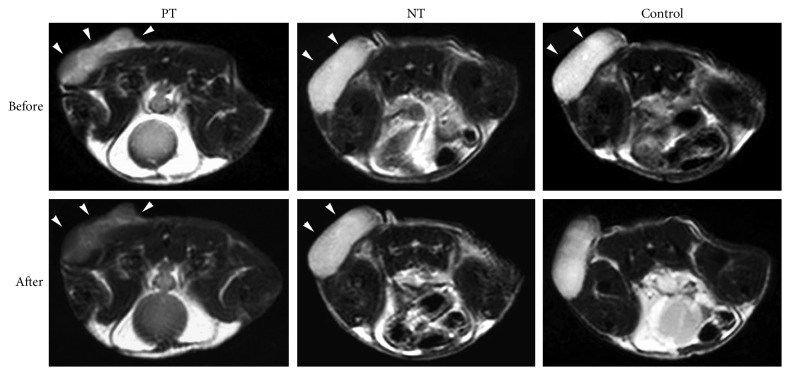
In vivo MR imaging of GPC3-expressing tumors via L5 peptide-guided two-step pretargeting approach. The tumor cells were implanted subcutaneously in the back of animals. On 1 hour postinjection T2WI image, xenografts (arrowheads) showed significant signal decrease in the pretargeting group (PT) but not in the nonpretargeting (NT) or control group.

A total of 8 subcutaneous tumor nude mouse models were established. The postinjection MR imaging (Figure 7) of xenografts showed significant signal intensity decrease on T2WI in the pretargeting group, with an enhanced ratio of about 32%, in contrast to an enhanced ratio of approximately 6% in both nonpretargeting group and control group. The difference was statistically significant (*P* < 0.001). Muscle tissue showed negligible enhancement in all three groups (Table 1).

### 3.7. Prussian Blue Staining and Histologic Examination

**Figure 8:**
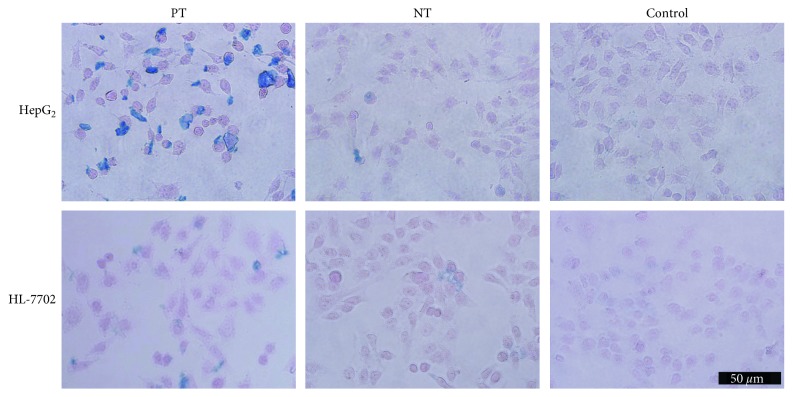
Prussian blue staining. Rich deposition of iron oxide particles (blue) was found in the pretargeting group (PT) of HepG_2_ cells, compared to the nonpretargeting (NT) or control group.

**Figure 9:**
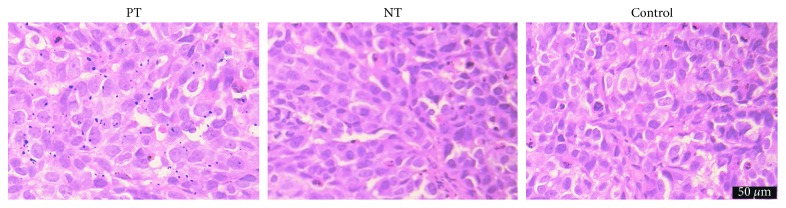
Prussian blue staining of xenografts. A large amount of blue granules were noted in the pretargeting (PT) group but not in the nonpretargeting (NT) or control group.

**Figure 10:**
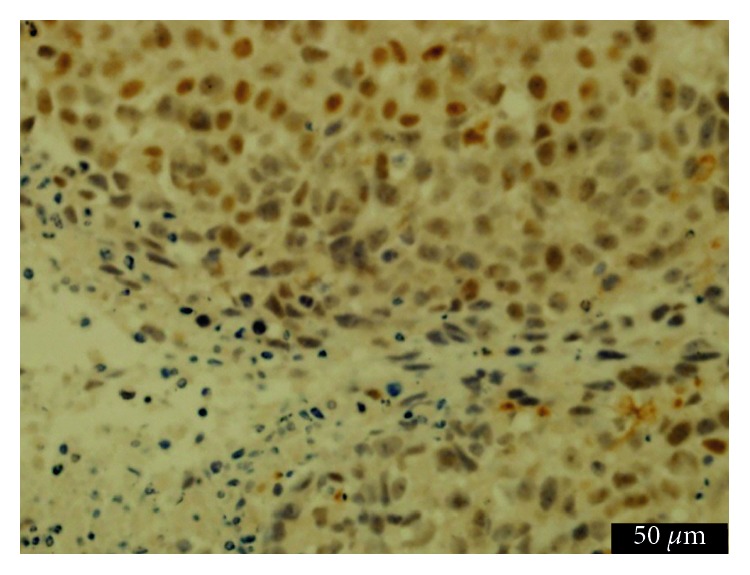
Immunochemistry staining of xenografts. Brown granules were noted on the surface and in the cytoplasm of most HepG2 tumor cells.

In vitro, numerous blue granules were found in HepG_2_ tumor cells in the pretargeting group, in contrast to little or no blue granules in the nonpretargeting group or control group (Figure 8). For in vivo studies, marked iron deposition was found on the surface and in the cytoplasm of tumor cells only in the pretargeting group (Figure 9). IHC staining showed that most HepG2 cells expressed GPC3 protein (Figure 10). These demonstrated the specific uptake of the probe by tumors via the binding of streptavidin and L5 peptide pretargeted on GPC3 antigen of HepG_2_ cells.

## 4. Discussion

Early diagnosis of hepatocellular carcinoma (HCC) is crucial to defining the appropriate course of treatment and prognosis of patients with the malignancy. Current methods used to detect and diagnose HCC are not optimal, specifically for small tumors, leading to a delay in diagnosis and treatment. In the present study, we demonstrated the potential of L5 peptide to serve as a specific ligand to guide magnetic NPs to GPC3-expressing HCC, as well as a way to intensify the signal, through two-step pretargeting approach.

As an emerging molecular target for HCC, GPC3 has attracted increasing attention in the past decade [10, 14, 25, 26]. Anti-GPC3 moAb and its F(ab')2 fragment have proven to be effective tools in enabling tumor-specific diagnosis through their ability to deliver imaging probes directly to the GPC3 receptor [11, 15, 26, 27]. Peptides have several advantages over moAb, in that they are easy to synthesize and generally do not present with immunogenicity [17]. In a recent study by Lee's group, L5 peptide was shown to have strong affinity and high specificity for GPC3 [22]. In agreement with the findings of Lee et al., direct immunofluorescence imaging and competitive binding assays in the present set of experiments demonstrated specific binding of L5 ligand to GPC3 (Figure 2). Moreover, both in vitro and in vivo MR imaging showed a pronounced enhancement of imaging in the pretargeting HepG_2_ group (Figures 6 and 7). The specificity of L5 peptide for GPC3 was further demonstrated by histologic examination (Figures 8 and 9). Of note, the signal intensities decreased by approximately 20% in vitro and 6% in vivo in the control groups, which might be caused by a nonspecific interaction between SA-USPIO-PEG particles and tumor or hepatic cells [26]. These experiments not only confirmed specificity of L5 to GPC3 but also confirmed that this interaction could be exploited to enhance MR imaging of HCC.

Iron oxide-based contrast agents are widely used in the field of molecular MR imaging because of their favorable properties such as superparamagnetism and safety [28]. Given the large amount of Kupffer cells in the liver capturing and eliminating extraneous particles, it is necessary to modify the surface of NPs to optimize the delivery efficiency of USPIO to their cellular targets [7]. Incorporation of PEG helps overcome biologic delivering barriers, increase access to targeted molecules and improve the biocompatibility of NPs [7, 29, 30]. In the present study, USPIO was coated with PEG and functionalized with SA; the resultant SA-USPIO-PEG maintained a high T_2_ relaxivity (Figure 4), apart from showing low toxicity (Figure 5) and a negative zeta potential (Figure 3(f)). The negative surface charge allows deeper tissue penetration of SA-USPIO-PEG to the target by minimizing nonspecific binding to surrounding tissues [31]. Also, the hydrodynamic size of nanoparticles is of importance and should be maintained between 10 nm and 100 nm [7]. In the present study, the mean hydrodynamic size of SA-USPIO-PEG was about 36 nm (Figure 3(d)). This would enable extravasation of the NPs from leaky tumor vessels and accumulation in tumor cells via enhanced permeability and retention, while avoiding quick renal clearance at the same time [27, 32]. In short, SA-USPIO-PEG prepared in this study had superparamagnetism, good biocompatibility, and desirable physical properties.

One drawback to this general approach is that peptides have lower avidity to targeted molecules than do antibodies, owing to their smaller molecular sizes [16]. This would have a negative influence on the sensitivity of molecular imaging. The strategy based on the biotin-avidin system, either a two-step or three-step protocol, is a versatile method to amplify signal intensity and thus was employed in the present study to handle the potentially lower amount of NPs uptake by tumor cells [24, 33]. In this preliminary study, we chose the less complicated two-step pretargeting protocol. The biotinylated L5 peptide was administered first to bind GPC3 on tumor cells (pretargeting), and then, SA-NPs were administered to chase the biotinylated L5 peptide. Our results indicated that the two-step protocol is feasible with biotinylated L5 peptide as a reporter molecule for HepG2 cells and USPIO-PEG as the contrast agent.

Owing to the very high specificity of GPC3-targeted approach [10], this method might be incorporated into daily clinical practice in the future. Biotinylated L5 peptide shall be administrated 12–24 hours prior to MR imaging, and USPIO-enhanced T2WI is recommended to be acquired 1-hour postinjection of SA-PEG-USPIO after completion of all other sequences. Although this method is logistically inconvenient, it can provide important information not obtainable from currently clinically used modalities, such as conventional MRI and MR imaging with Gd-DTPA, Gd-BOPTA or Gd-EOB-DTPA, which will help improve the diagnostic performance of MR imaging for HCC. For instance, hepatic dysplastic nodule, adenoma, and cavernous hemangioma, as well as liver metastasis without known primary tumor, express little or no organic anion-transporting polypeptide (OATP) and thus may resemble HCC on hepatocyte-phase images, especially those subtypes of high differentiation expressing a little OATP. Also, the difference of OATP expression is just about 60% between normal hepatocytes and HCC cells and dependent on the liver function, which may further compromise the diagnostic performance of MR imaging with Gd-EOB-DTPA in the setting of liver cirrhosis [34]. In addition, Gd-EOB-DTPA-enhanced MR imaging may be of limited use for the assessment of HCC nodules with diameter of less than 20 mm [35].

Several limitations of the present experiments should be noted. First, we have shown proof of principle in terms of cellular and molecular biology and verified the feasibility of this approach to detect subcutaneous xenograft of GPC3-expressing HCC. However, whether this strategy will sufficiently aid with visualization of orthotopic tumors needs to be investigated. It is possible that HCC foci might be shadowed on superparamagnetic iron oxide nanoparticle- (SPION-) enhanced T2WI because surrounding liver parenchyma may show SPION enhancement along with HCC lesions. However, even if this did happen, we can still locate HCC lesions using other MR sequences (including precontrast T2WI). Thus, the signal decrease produced by SPION uptake can be calculated by comparison between precontrast T2WI and postcontrast T2WI, through which we can specifically evaluate the nature of the lesion. Second, the comparison between an anti-GPC3 monoclonal antibody and L5 peptide in terms of their efficacy to guide USPIO probes to tumor cells was not assessed. On the one hand, L5 peptide has lower avidity to GPC3 antigen than does anti-GPC3 monoclonal antibody, owing to its smaller molecular size. On the other hand, NPs coupled with antibody, compared to those linked with L5 peptide, is more readily to be cleared by macrophages in vivo and has poorer tissue penetration, which may decrease the delivery efficacy of antibody-based targeting approach. Moreover, L5 peptide-mediated approach used in our report took advantage of the amplification effect from the biotin-avidin system, which can augment the efficiency of delivering NPs to tumor target. Therefore, it is reasonable to speculate that both strategies might produce comparable contrast enhancement of HCC lesions in orthotopic model.

## 5. Conclusion

In summary, USPIO-based imaging probe with superparamagnetism and low cytotoxicity was synthesized, and the feasibility of the L5 peptide-mediated two-step pretargeting approach to specifically identify GPC3-expressing HCC was validated using both in vitro MR imaging and in vivo MR imaging. This detection method may be useful in the early detection and diagnosis of HCC and other targetable cancers.

## Figures and Tables

**Table 1:** Enhanced ratio (%) of tumor and muscle from in vivo MR imaging.

	Pretargeting (*n*=8)	Nonpretargeting (*n*=8)	USPIO (*n*=8)	*P* value
Tumor	31.8 ± 0.5	5.6 ± 0.5	6.1 ± 0.7	0.000
Muscle	4.6 ± 0.4	4.7 ± 0.3	4.9 ± 0.6	0.572

## Data Availability

The data used to support the findings of this study were provided by General Electric Company under license and so cannot be made freely available. Access to these data will be considered by the author upon request, with the permission of General Electric Company.
